# Cervical cancer in Iran: integrative insights of epidemiological analysis

**DOI:** 10.1051/bmdcn/2018080318

**Published:** 2018-08-24

**Authors:** Zohre Momenimovahed, Hamid Salehiniya

**Affiliations:** 1 Qom University of Medical Sciences Qom Iran; 2 Zabol University of Medical Sciences Zabol Iran; 3 Department of Epidemiology and Biostatistics, Tehran University of medical sciences Tehran Iran

**Keywords:** Cervical cancer, Epidemiology, Prevention, Risk factor, Iran

## Abstract

Background: Cervical cancer is a common cancer among women around the world. Due to the geographical differences in incidence, mortality and risk factors of cervical cancer, it is necessary to conduct different studies in different countries. This review study is aimed to investigate the most important aspects of cervical cancer in Iran.

Methods: Reviewing papers without time limitation was conducted with the keywords cervical cancer, Cervix uteri cancer and Iran in their title or abstract. The databases of Medline, IranMedex, SID, ScienceDirect, Embase, Google Scholar, Web of Science, and Scopus were searched. The title and abstract of the papers were reviewed, in all, 51 full papers were reviewed.

Results: Based on published studies, the incidence of cervical cancer varies between different areas of Iran. The findings of this study demonstrated that there is a relationship between marital status, marriage age, and age of first pregnancy, smoking, and consumption of oral contraceptive pills, multiple sexual partners, family history, multiparty, and cervical cancer.

Conclusion: The results of this review showed that the incidence of cervical cancer in Iran is low; however, the risk factors associated with this cancer are not few, which could lead to the increase in the incidence of cervical cancer in the future.

## 1. Introduction

Cervical cancer is a common and deadly cancer among women around the world [1]. According to global estimates, about half a million new cases are discovered yearly in the world, most of which are in developing countries [2, 3]. With the increase in high-risk sexual behaviors, this cancer is anticipated to increase [4]. Cervical cancer is on the list of preventable cancers, since it can be prevented, as due to its long precancerous conditions, screening programs [5, 6], as well as effective treatment of precancerous lesions [7] are available. In most cases, cervical cancer is caused by the slow progression of precancerous lesions, and therefore, at this time, abnormal cells are detected with Pap test [8]. Several risk factors lead to cervical cancer, the most important of which are biologic, socio-economic and health factors [9]. Many epidemiological studies have pointed out the role of sexually transmitted infections [10], reproductive [11], behavioral [12] and nutritional factors [13] involved in the incidence of the cancer, among which the infection with one type of the human papillomavirus (HPV) oncogene is the most important and vital cause [14, 15]. Despite the fact that many years have passed since the introduction of pap test, this cancer is still a cancer-related cause of morbidity all over the world [1].

**Table 1:** ASR of cervical cancer in Iran.

Row	First author	Year	Place of study	ASR (per 100000 persons)	Source
1	Arab	2005	National cancer registry of Iran	1.4	(24)

2	Talaiezadeh	2002-2009	Khuzestan	2.56	(30)

3	Arab	2004-2008	National cancer registry of Iran	1.7 in 2004 to 2.2 in 2008	(25)

4	Fateh	2001-2010	Shahroud	1.8	(31)

5	Khorasanizadeh	2013	Analyses of national data and systematic review	2.5 in pathology-based cancer registry 6 in population-based cancer registry	(23)

6	Taheri	2004-2010	Golestan population-based cancer registry	6.22 in city and 3.77 in village (total: 4.97)	(32)

7	Mohagheghi	1998-2001	Tehran population-based cancer registry	4.8	(33)

8	Babaei	2004-2006	Ardabil population-based cancer registry	1.4	(34)

9	Chaichian	2003-2009	National cancer registry	1.64 in 2003 to 2.17 in 2009 In 2009, highest incidence was in Yazd (7.4), Isfahan (5.9) and Tehran (5.84) Lowest incidence was in Kohkiluye-Boyer Ahmad	(35)

**Table 2:** Cervical cancer risk factors in Iran.

Row	First author	Year	Place of study	Type of study	Number of participants	Risk factor(s)	Source
1	Vaisy A	2013	Tehran	Case-control	128 patients 128 controls	Marital status (divorced or widowed) Married more than once Marriage at age below 16 Contraceptive pill consumption Protective factor: Cryotherapy	(36)

2	Nojomi M	2006	Tehran	Crosssectional	300 patients	Positive family history of cervical cancer Low marriage age High prevalence of pregnancy Low age at first pregnancy Prolonged consumption of contraceptive pills	(37)

3	Boheirayi A	2006	Tehran	Case-control	109 patients 218 controls	Protective factor: use of condom	(42)

4	Mohaghegh F	2015	Arak	Crosssectional	1000 women	Multiple marriages Being widowed or divorced Multiple sexual partners ¬There was no significant relationship between smoking, diet, consumption of pregnancy pills with cervical cancer	(43)

5	Karimi Zarchi M	2010	Yazd	Crosssectional	100 patients	Low-age marriage Multi parity Pow age at first pregnancy (16.2 years) Smoking Not doing Pap smear	(38)

6	Vaisy A	2014	Tehran	Case-control	128 cases 128 controls	Marital status Marriage more than once Marriage before 16 Sexual intercourse more than twice a week ¬Sexual intercourse during menstrual period did not significantly correlate with cervical cancer.	(41)

7	Dehaghani AS	2002	Shiraz	Case-control	23 patients with cervical epithelial cell carcinoma 36 individuals for control	Existence of HLA-DQB1*0601	(44)

8	Taherian AA	2001	Esfahan	Case-control	87 patients 195 controls	Age at first marriage (P=0.0008, OR=5) Number of deliveries (P=0.0001, OR=3.2) Contraceptive method (P=0.008, OR=3.3)	(39)

9	Vaisy A	2014	Orumieh	Case-control	128 patients 128 controls	Use of contraceptive pills (this relationshipis significant with the duration of theconsumption).	(40)

10	Jafari M	2007	Tabriz	Crosssectional	6024 individuals	Number of deliveries of 3 cases and more Number of pregnancies of 3 cases and more Number of abortions of one case and more Multiple sexual partners Protective factor: use of condom	(45)

**Table 3:** Knowledge, attitude and performance about cervical cancer in Iran.

Row	First author	Year	Place of study	Type of study	Number of participants	Main findings			Source
						Knowledge	Attitude	Performance	
1	Jalalvandi M	2006	Arak	Cross sectional	778 individuals	44.5% of the samples were not aware of the existence of Pap smear for early diagnosis of cancer.	-	17.5 % of the samples performed the screening withregular intervals. There was a relationship between education and age with screening performance (*P* < 0.001).	(58)

2	Farzaneh F	2011	Tehran	Cross sectional	500 individuals	More than half of the participants were not aware of HPV, its transmission ways and prevention of cervical cancer.	The most common concern of the subjects was about the side effects, efficacy and cost of the HPV vaccine.	-	(59)

3	Javanmanesh F	2008	Tehran	Cross sectional	1002 individuals	77.9% of the individuals knew about the possibility of cervical cancer discovery using Pap smear. 44.1% of the individuals were aware about the time that the first Pap smear should be done.	-	-	(57)

4	Asgarlou Z	2014	East Azarbaijan	Cross sectional	600 students 400 hospital and university staff	29.1% of the individuals did not have any information about cervical cancer.	-	-	(60)

5	Karimi Zarchi S.M 2013	2013	Yazd	Cross sectional	380 nurses	36.7% of the samples were aware of the HPV infection and its association with cervical cancer. 58.3% of the samples were aware about the HPV transmission through sex, and 11.2% of them were aware about the transmission of this virus by skin contact.	41.2% of the samples were eager to use the vaccine and the rest were reluctant to use the vaccine; where 26.2% was due to inadequate knowledge about the vaccine and 41.4% was due to concerns about vaccine side effects.	-	(61)

6	Rezaie-Chamani S	2011	Rasht	Cross-sectional	350 individuals	The mean awareness score was 59.4 in the possible range of 0-100.	40.9% of women believed that in the absence of symptoms, Pap smear is unnecessary. There was a significant positive correlation between knowledge and attitude (*p* = 0.01).	27.1% of the samples did at least one Pap smear.	(62)

There is a high geographical variation in the incidence and mortality of cervical cancer which can be attributed to socioeconomic differences [16] and various causes such as low access to screening, not doing prevention programs, ineffective and inadequate treatment, and poor health conditions [16-18]. The incidence of cervical cancer is small in Iran and in many Muslim countries, although its mortality is remarkable [19]. Due to the geographical differences in incidence, mortality and risk factors of cervical cancer, it is necessary to conduct distinct studies in different countries. Many studies have been conducted on cervical cancer in Iran, each of which has dealt with one of its aspects [20-23]. Considering the lack of a comprehensive study on this cancer as the aspect of epidemiology in Iran, this review study is aimed to investigate the most important aspects of cervical cancer including incidence, mortality, risk factors, prevention programs and screening, knowledge, attitudes and performance of women regarding the cancer and the ways of screening and its prevention in Iran.

## 2. Methods

Reviewing papers without time limitation was conducted in order to investigate incidence, mortality, risk factors, and also screening and diagnosis of cervical cancer in Iran. The search strategy included all papers with the keywords cervical cancer, Cervix uteri cancer and Iran in their title or abstract. Other unpublished papers were also searched manually. The databases of Medline, Science Direct, Embase, Scopus, Google Scholar, Web of Science, Iran medex and SID were searched. The title and abstract of the papers were reviewed. The papers that investigated various aspects of cervical cancer in Iran were included in the study. The papers that addressed the therapeutic and surgical aspects of cervical cancer were excluded.

## 3. Results

### 3.1. Study characteristics

In the initial electronic literature search, 396 articles were obtained from databases and 12 articles were obtained using manual search. After removing duplicates using Endnote X7 (n = 104), the title and abstract of the remaining 304 articles were reviewed. After this stage, 95 articles were included in the study and 44 of these articles were removed because of scientific reasons and lack of eligible criteria, in all, 51 full papers were reviewed.

### 3.2. Incidence and mortality

In 2005, Arab and colleagues reported age specific incidence rate (ASR) of cervical cancer was 1.4 in 100000 individuals, which reached its peak in the ages of 60-64 years with 6.8 [24]. The results of one study showed that ASR for cervical cancer increased from 1.7 in 2004 to 2.2 in 2008. They reported an increase in the incidence of cervical cancer in this study [25], although its incidence is still the lowest compared to many countries [26-29]. Talaiezadeh *et al.* stated that according to the data of the Khuzestan Cancer Registry Center, in 2002-2009, the ASR of cervical cancer was reported 2.56 [30]. The analysis of cancer data in Shahroud in 2001-2010 showed the ASR of cervical cancer to be 1.8. Cervical cancer was more common in younger women in this study [31]. According to Globocan (2012), ASR for cervical cancer in Iran is 2.8 and mortality is 1.2 in 100000 individuals [19]. Khorasanizadeh *et al.* in 2013, analyzed the National Cancer Registry data, and stated the average ASR of cervical cancer in Iran is 2.5 , where the highest was in Fars (4.1) and the lowest was in Zanjan (0.4) [23]. In 2014, Taheri *et al.* analyzed the data from the Cancer Registry Center in Golestan and introduced cervical cancer as the second most common gynecological cancer, after ovarian cancer, and stated the ASR of cervical cancer as 6.22 in the city and 3.77 in the village [32]. Analysis of the data from the Cancer Registry Center in Tehran showed that the ASR of cervical cancer was 4.8 [33]. This figure was 1.4 in Ardebil [34], 4.16 in East Azerbaijan, 5.43 in Markazi, 7.14 in Yazd and 5.99 in Isfahan [35]. The incidence of age-specific cervical cancer peaked at 60 years old [23]. The range of the ASR of cervical cancer was different, from 0.4 in Zanjan to 6.22 in Golestan. In 2012, 370 deaths from cervical cancer were registered in Iran and it is anticipated to increase by 2035 in all age groups [4]. The ASR for cervical cancer mortality in 2012 was 1.2 [4]. In a review study, the ratio of mortality to cervical cancer incidence was 42% [23] (table 1).

### 3.3. Risk factors

Several studies focused on the role of risk factors in the incidence of cervical cancer in Iran. These studies are listed in Table 2. The results of these studies generally show that there is a relationship between marital status [36], marriage age [36-39], age of first pregnancy [38], smoking [38], consumption of oral contraceptive pills [36, 37, 40], multiple sexual partners [36, 41], family history [37], multi parity [37], and cervical cancer. However, there were contradictions in the results of the studies. For example, there was no relationship between oral contraceptive pills and cervical cancer in the study by Bahiraie *et al.* [42].

### 3.4. Human Papillomavirus, the most important risk factor

Human papillomavirus is a DNA virus [21]. Many studies have pointed out the causal role of the Human Papillomavirus as the major risk factor for cervical cancer [46]. The E6 and E7 proteins of the virus affect cell cycle. In addition, the integration of the viral genome in host DNA causes excessive expression of E6 and E7 proteins, which disables P53 proteins and retinoblastoma of host cells. Due to the association of some species with cervical cancer as well as the ability to merge into the host genome, some virus species are called high-risk [23].

### 3.5 Prevalence of HPV in Iran

Prevalence of HPV is diverse in terms of geographical region [47]. In their study Allameh *et al.,* proved that 90.8% of HPV was found in cervical cytology [48]. However, in the study by Sadeghi *et al.,* HPV DNA was found in 30.7% of the cervical carcinomas [49]. In the meta-analysis conducted by Jalilvand, the overall prevalence of HPV in the high-grade squamous intraepithelial lesions was 79%. This rate was 62% in low-grade intraepithelial lesions. Among normal people, HPV was seen in 9% of them, varying from 5.5% in the south to 13.6% in the north of the country [47]. In the study by Khodakarami, HPV was estimated to be 7.8% in the general population, which is lower than other countries [50]. In a study of Shiraz, 87.1% of cervical cancer patients were HPV-positive in terms of DNA [21]. In the study by Mortazavi *et al.* in 2002, 73.9% of HPV-positive tumors were type 16 and the rest were type 18 and 31. In this study there was no relationship between tumor histology and HPV type [51]. The survey of national cancer registries and a systematic review of Iranian studies showed that 76% of patients with cervical cancer were HPV-positive. The most common type of HPV was type 16 (56%), type 18 (15%), and type 31 (10%). According to the results of this study, the prevalence of HPV in the general population of women was 7% [23]. Another study reported the HPV DNA in 5.5% of healthy subjects, and in 2% of cases, its type was proven high-risk. Although the overall HPV prevalence was higher in higher ages and peaked at 50-59 years old in this study, the high-risk types of the virus were found only in younger women [52].

HPV genotypes vary by geographical region. The results of a meta-analysis indicated that 6 common types of HPV, *i.e.* types 18, 16, 11, 6, 31, and 33 were in invasive cancer lesions, high-and low-grade intraepithelial lesions, and atypical squamous cells. Six common types of 16, 18, 6, 11, 31, and 45 are seen in normal people. The most common type of HPV was type 16 in squamous cell carcinoma lesions, followed by type 18. However, they were the same in adenocarcinoma [47].

### 3.6. Screening

The National Screening Schedule in Iran was implemented by the Ministry of Health and Medical Education in 1989 [53]. Access to appropriate screening schedules and the availability of national guidelines can lead to early detection and timely treatment. Khodakarami *et al.* recommended starting the cervical cancer screening at the age of 30 with a repetition of every five years and continuing until the age of 69 [54]. The results of a study showed that the most cost-effective screening method is screening with HPV that begins at the age of 35 and is repeated every 10 years [55]. Nokiani *et al.* conducted a study to determine the cost-effectiveness of Pap smear in Kermanshah. They concluded that due to the lack of recognition of HSIL or carcinoma before the age of 35, and regarding the fact that, in order for LSIL to change to HSIL or carcinoma, more than 5 years is needed, performing Pap smear before age 35 year is not effective in Iran [56].

### 3.7. Knowledge, attitude and performance of Iranian women about cervical cancer, screening and vaccination

Awareness is one of the most important predictors of health behaviors and effective factors in performing screening methods. But awareness alone is not enough, because the attitude is also a major factor in preventive behaviors. In addition, cancer control performance would not succeed without a positive attitude [57]. The results of studies in this field are shown in Table 3. As the results of the table show, Iranian women do not have the desired level of knowledge, attitude and performance.

### 3.8 Screening barriers and facilitators

Cervical cancer screening for early detection is one of the most important ways cancer prevention that can lead to timely cervical cancer treatment, followed by increased life expectancy of the patient. However, different factors can affect the patient’s behavior in screening for cervical cancer. Several factors in the text of the studies have been identified as barriers to screening, among which the most important are the lack of awareness about cervical cancer and its screening methods [63-65], the lack of financial support and the cost of testing [62, 63, 65], as well as the lack of information support [62, 63, 66]. Social norms are one of the most important determinants of performing the screening [67]. Shame and embarrassment are important barriers to screening for cervical cancer that prevent a person from the timely performing of screening [66].

Cervical cancer screening is facing many challenges. According to the results of a qualitative study, lack of awareness, inappropriate attitudes, cultural taboos, sociocultural barriers, immoral relationships, systemic defects, poor intersection collaboration, poor management, and insurance are among the most important challenges for cervical cancer screening [68]. Financial issues, fear of test results and lack of awareness [69], lack of free time, absence of symptoms [64], depression, tiredness, and poor quality of services provided from the view of patients [65], incorrect beliefs, process-related pain [63], and belief in the unavoidable nature of cancer [64] are mentioned as barriers to screening in various texts.

On the one hand, education can increase people’s knowledge about cervical cancer, change their attitude and encourage them to perform Pap test after the reviewing the literatures[70]. In this regard, the role of service providers as awareness-giving individuals is very high in encouraging people to perform screening [64]. Akbari et al. mentioned the incentives imposed by service providers and perceived risk as accelerators of screening [64]. Understanding the benefits of performing Pap smear can act as one of the most important motives for screening for cervical cancer [20]. In a qualitative study, Shakibazadeh referred to addressed doctor’s counsel, relatives’ and families’ advice, knowledge about the symptoms and screening methods, free and accessible service and risk perception as being among the most important factors that encourage a person to perform screening [63].

### 3.9. Histology and survival rate

Neoplastic changes in Pap smear results in Iran are low [71]. In the study by Mortazavi *et al.*, 87% of cervical cancer cases were squamous cell carcinoma and the rest were adenosquamous and adenocarcinoma [51]. In their study, Sadeghi *et al.* reported 73% squamous cell carcinoma, 7.6% adenocarcinoma, 11.5% poorly differentiated carcinoma and 7.6% carcinoma in situ (CIN III) [49]. In a study in Kermanshah, the incidence of HSIL and carcinoma was determined as 26.9 in 100000 individuals [56]. The results of a study showed that the survival rate of squamous cell carcinoma is 75% and the survival rate of adenocarcinoma is 87.5% [72].

### 3.10. Prevention

Cervical cancer is the most common known malignancy associated with the Human Papillomavirus; this fact led to the introduction of a vaccine against the virus. In order to prevent cervical cancer, the design of a national instruction for the organization of cervical cancer screening schedules as well as the design of patients’ screening protocol, including the follow-up and management of patients with precancerous lesions and patients with cervical cancer, are among the most important priorities [73].

Khatibi *et al.* conducted a study on the cost-effectiveness of a four-capacity vaccine, and concluded that HPV vaccination in Iran is not cost-effective [74]; however, with an increase in the prevalence of cervical cancer, this conclusion may become inaccurate. The use of the vaccine is also one of the variables that indicate its acceptability. In the study by Mojahed *et al.,* concerns about vaccine safety, the high cost of the vaccine, and lack of adequate knowledge about the vaccine and its potential side effects were mentioned among the reasons for reluctance to vaccinate against HPV [61].

## 4. Conclusion

This study was conducted for a comprehensive review as the aspect of epidemiology of the texts related to cervical cancer in Iran. Although the incidence of cervical cancer in Iran is low in comparison with other geographical areas, the risk factors associated with this cancer are not few, which could have led to the increase in the incidence of cervical cancer in recent years, and hence requires special attention. This study can be used as a guide for future studies as well as the design of national cervical cancer schedule in Iran.

## Conflicts of interest

The authors declare no conflict of interest.
